# DESPITE OUR BEST EFFORTS, THEY AREN’T BUYING WHAT WE’RE SELLING: RAISING HARD QUESTIONS ABOUT OUR FUTURE

**DOI:** 10.1093/geroni/igae098.1037

**Published:** 2024-12-31

**Authors:** Julie Masters

**Affiliations:** University of Nebraska, Lincoln, Nebraska, United States

## Abstract

In the 1970s, funding from the Older Americans Act was made available to establish gerontology programs at universities and colleges in anticipation of a growing aging population. The baby boom that led to early education programs and the building of schools and training of teachers was the driver behind visionaries who knew these same people would one day benefit from gerontological insights. Fast forward to 2024: 12,000 people are turning sixty-five each day. The long-expected demographic shift is here. The workforce to support the aging population, however, has fallen short, with demand outpacing supply in the public and private sectors. Nonetheless, many of the educational programs designed to prepare a well-prepared and sufficient workforce are struggling amidst a backdrop of academic budget cuts, revenue shortfalls, and the perennial issue of inadequate student/practitioner interest (Gurwitz, 2023). Hard questions must be asked if gerontological and geriatric education is to have a strong and sustainable future. This session poses several of these questions, examining such issues as branding (e.g., Gerontology Vs. Aging Studies), stand-alone and integrated delivery of educational programs, alliances (e.g., academic, community, corporate), preparation and credentialing (e.g., degrees, micro-credentialing, internships, practicum, on-the-job training), and career value (e.g., job types, status, compensation). The session concludes with the question, if not gerontology and geriatrics, who will be the champion for older adults?

